# The relationship between education and health among incarcerated men and women in the United States

**DOI:** 10.1186/s12889-016-3555-2

**Published:** 2016-09-01

**Authors:** Kathryn M. Nowotny, Ryan K. Masters, Jason D. Boardman

**Affiliations:** 1 Department of Sociology, University of Miami, 5202 University Drive, Coral Gables, FL 33146 USA; 2 Department of Sociology & Institute of Behavioral Science, University of Colorado Boulder, 327 UCB, Boulder, CO 80027 USA

**Keywords:** Education-health association, Education, Morbidity, Gender, Prisoners, Prison

## Abstract

**Background:**

This paper contributes to research on the education-health association by extending the scope of inquiry to adult inmates. Not only are inmates excluded from most nationally representative studies of health but they also represent a highly select group in terms of both education and health. As such, our study provides new information about the health of incarcerated populations and it extends the generalizability of the education-health association beyond the non-institutionalized population.

**Methods:**

We use a prison-level fixed-effects regression model with the 2004 Survey of Inmates in State Correctional Facilities (*n* = 287 facilities) to evaluate the effects of education on a standardized morbidity scale of 11 lifetime and current health conditions among incarcerated men (*n* = 10,493) and women (*n* = 2,797).

**Results:**

Education prior to incarceration is negatively associated with lifetime health problems for both women and men and the association is stronger among women. Among inmates who enter prison with less than a GED level of education, attaining a GED in prison is associated with better current health outcomes for men, but not women.

**Conclusions:**

The generalization of the education-health association among prisoners further highlights the fundamental nature of education as a health promotive resource. Discussed are the implications for the education-health literature in general and health promotion efforts among incarcerated adults specifically.

## Background

This study examines whether level of education prior to imprisonment and participation in prison education programs are associated with better health for male and female inmates. We contribute to a large body of work linking educational attainment and adult health [1–3] in which individuals with higher levels of education report fewer chronic diseases, have better mental health, and enjoy longer lives than do adults with lower levels of education [4, 5]. The resource substitution theory hypothesizes that education will be more strongly associated with health among more disadvantaged populations, such as women relative to men, because education can serve as a “substitute” for the limited resources among these groups [6, 7]. However, because of the explicit focus on non-institutionalized populations [8], little is known about the health benefits of education among male and female prisoners.

The absence of this research is particularly important because the size of the prison population in the United States has increased more than sevenfold in the past 30 years and the U.S. now has the highest rate of imprisonment compared to all other countries [9]. Importantly, the female prison population has been increasing at substantially higher rates than the male prison population [10], incarcerated women have worse health [11] and complex comorbid conditions [12] across multiple domains compared to men. In addition, women’s prisons have lower programming availability [13], often struggle to meet the healthcare needs of the prisoners [13, 14], have higher exposure to sexual violence [15], and women have specific healthcare needs that are not always provided for in prisons [16, 17]. As such, examining sex differences in the relationship between education and health among prisoners is important because it provides additional insights into the nature of the association.

## Methods

Data come from the 2004 Survey of Inmates in State Correctional Facilities (SISCF), which provides a nationally representative sample of U.S. adults incarcerated in state prisons [18]. The sample design employed a stratified, two-stage selection process. The prison sample was selected from a universe of 1,585 state prisons. Overall, 301 prisons were randomly selected for inclusion in the study. A total of 287 prisons participated (95.3 % prison-level response rate). In the second stage, inmates were randomly selected for participation (*n* = 14,499; 89.1 % respsonse rate). The interviews were conducted using computer assisted personal interviewing and participation was voluntary. Due to missingness on key variables, the final sample size is 13,290 (10,493 men and 2,797 women).

Respondents were first asked about *lifetime* diagnoses and then a follow up question ascertained whether or not the respondents still have problems related to the condition (i.e., *current* morbidity). The 11 health conditions include hypertension or high blood pressure, diabetes, heart problems, asthma, kidney problems, stroke, all-cause cancer, arthritis, cirrhosis, sexually transmitted infections (STI), and hepatitis. Lifetime (α = 0.52) and current (α = 0.48) morbidity standardized scale scores are calculated to represent the presence of multiple health conditions. Education prior to entering the current prison term is measured with years of education (0 to 18 years). We further refine this measure by including a series of dummy variables assessing degree attainment by categorizing less than high school, GED, high school diploma, and at least some college. Current education examines whether an inmate participated in a high school education class or obtained a GED during their current prison sentence if they are eligible (i.e. enter prison with less than a GED level of education). All multivariate models control for age, race/ethnicity, employment prior to incarceration, and marital status. We also control for the number of years served to date during the current incarceration episode and whether this is their first incarceration episode.

Our analyses are conducted in two parts. First, linear regression models estimate the association between education *prior to entering prison* and *lifetime* morbidity stratifying by gender and controlling for demographic background. Importantly, our models use prison fixed effects to control for contextual and compositional differences across prisons that may confound associations. The second analysis examines educational attainment within prison and includes only those inmates who entered prison with less than a GED level of education. Similar linear models with prison fixed effects estimate the association between attaining a GED *in prison* and *current* morbidity. We then follow with additional descriptive analyses to examine who participates in prison high school classes in more detail. Finally, a supplementary analysis examines the association between education and one of the morbidities: hypertension. All descriptive and multivariate analyses use sampling weights to adjust for the complex sampling design of the study.

## Results

Table 1 presents the descriptive and bivariate (by gender) analysis. Incarcerated women have a significantly higher mean morbidity count than do men. Half of women (52.8 %) report at least one health condition while only one-third of men do (36.9 %). The top 10 % of the unhealthiest women have three current health conditions compared to two for men. The raw frequencies and weighted odds ratios indicate that women have worse health compared to men. Incarcerated women report significantly higher rates for most conditions at the *p* < 0.001 level excluding hypertension - which is still significant at the *p* < .01 level - and stroke and cirrhosis - which are not statistically significant.

**Table 1:** Health, Education, and Demographic Characteristics of Prisoners Stratified by Gender (Men: *n* = 10,493; Women: *n* = 2,797)

	Men	Women	*p*	Weighted	Confidence
	*n* (%)^a^	*n* (%)^a^		OR	Interval (OR)
Mean Health Count (se)	.59 (.01)	.97 (.02)	< .001		
Hypertension	2197 (21.2)	649 (23.6)	< .009	1.16	1.04, 1.29
Heart Problems	964 (9.3)	317 (11.5)	< .001	1.32	1.14, 1.51
Diabetes	448 (4.3)	190 (6.9)	< .001	1.75	1.45, 2.11
Asthma	1495 (14.4)	620 (22.5)	< .001	1.78	1.60, 1.99
Kidney Problems	592 (5.7)	323 (11.7)	< .001	2.29	1.97, 2.65
Arthritis	1603 (15.5)	682 (24.8)	< .001	2.06	1.83, 2.32
Stroke	475 (4.6)	151 (5.5)	< .053	1.34	1.02, 1.76
Cancer	181 (1.8)	278 (10.1)	< .001	5.69	3.71, 8.73
Cirrhosis	175 (1.7)	49 (1.8)	< .746	1.48	0.97, 2.27
Hepatitis	972 (9.4)	432 (15.7)	< .001	2.20	1.85, 2.61
STI	1226 (11.8)	484 (17.6)	< .001	3.00	2.08, 4.31
Education Prior					
Mean Years (se)	10.4 (.02)	10.7 (.05)	< .001		
Less than GED	4334 (41.7)	1150 (41.4)	< .766	0.97	0.89, 1.06
GED	2615 (26.1)	555 (20.0)	< .001	0.74	0.66,0 .82
High School Diploma	2274 (21.9)	606 (21.8)	< .936	1.00	0.90, 1.12
Some College	816 (7.8)	328 (11.8)	< .001	1.60	1.39, 1.85
College	363 (3.5)	142 (5.1)	< .001	1.53	1.24, 1.90
Mean Age (se)	35.3 (.10)	35.6 (.17)	< .203		
Race/Ethnicity					
White	3901 (37.2)	1261 (45.1)	< .001	1.47	1.35, 1.61
Black	4652 (44.3)	998 (35.7)	< .001	0.67	0.61, 0.74
Latino	1369 (13.1)	352 (12.6)	<.518	0.87	0.77, 0.99
Other	571 (5.4)	186 (6.7)	< .014	1.31	1.09, 1.57
Employed Prior	7402 (71.0)	1586 (57.2)	< .001	0.55	0.50, 0.60
Marital Status					
Never Married	6134 (58.9)	1261 (45.2)	< .001	0.58	0.53, 0.63
Married	1576 (15.1)	505 (18.1)	< .001	1.24	1.11, 1.40
Widowed/Divorced/Separated	2762 (26.4)	1026 (36.8)	< .001	1.64	1.49, 1.80
Mean Time Served (se)	4.6 (.06)	2.5 (.07)	< .001		
First Time Incarcerated	4796 (46.4)	1629 (59.0)	< .001	1.67	1.53, 1.83

*OR* Odds Ratio, *CI* 95 % Confidence Interval, *se* Standard Error

^a^Raw sample size and frequency unless otherwise stated

Although the difference is small in magnitude, incarcerated women have a significantly higher mean years of education compared to men with men averaging 10.4 years of education and women averaging 10.7 years of education (Table 1). Patterns of degree attainment prior to entering prison also differ by gender. While similar proportions of men and women enter prison with less than a GED (41 %), men are significantly more likely to have earned a GED (26.1 %) than women (20.0 %). About 22 % of men and women obtained their high school diploma prior to their current prison stay. Women, though, have significantly higher rates of some college (11.8 %) and at least a four-year college degree (5.1 %) compared to men (7.8 % and 3.5 %, respectively).

Results from fixed effects (by prison) regression models examining education *prior to incarceration* and *lifetime* morbidity are presented in Table 2. The findings indicate that increases in years of education are negatively associated with the standardized lifetime morbidity scale for both incarcerated women and men, but that the effect is greater for incarcerated women.^1^ Considering that the comorbidity scale is standardized within each sex, it suggests that the effect of one year of additional education among women (*b* = −.017, *p* < .001) is more than four times the size of the comparable effect among men (*b* = −.004, *p* < .05). To further evaluate the magnitude of these effects, we standardized the education scores and the same four-fold comparison is evident (*β*_*women*_ = −.04, *se* = .010) and men (*β*_*men*_ = −.01, *se* = .004) but the effect sizes are clearly small. Degree attainment prior to incarceration is only significant for women. Interestingly, women with a GED (*b* = −.088, *p* < .001) have lower scores on the standardized lifetime morbidity scale compared to women entering prison with a high school diploma.

**Table 2:** Results from Regressing Level of Education Prior to Incarceration on Standardized Lifetime Morbidity Scale with Prison-Level Fixed Effect for Men and Women

	Lifetime Morbidity Scale							
	Men				Women			
	*b*	*p*	*b*	*p*	*b*	*p*	*b*	*p*
Education Prior to Incarceration								
Years of Education	−.004	*			−.017	***		
Education (High School Diploma Ref.)								
Less than High School			−.006				.036	
GED			−.010				−.088	***
At Least Some College			.002				−.036	
Age	.012	***	.012	***	.015	***	.015	***
Race/Ethnicity (White Ref.)								
Black	−.029	***	−.031	***	−.077	***	−.078	***
Latino	−.027	*	−.027	*	−.012		−.002	
Other	.067	***	.066	***	.047		.048	
Employed Prior to Incarceration	−.046	***	−.048	***	−.031	+	−.039	*
Marital Status (Never Married Ref.)								
Married	.022	*	.022	+	.038		.034	
Widowed/Divorced/Separated	.020	*	.020	*	.042	+	.040	+
Time Served to Date	−.001		−.001		.004		.004	
First time Incarcerated	−.015	+	−.017	*	−.069	***	−.069	***
Model Statistics								
Number of Inmates	9941		9914		2657		2656	
Number of Prisons	225		225		62		62	
Overall R Squared	.14		.13		.11		.11	
Rho	.042		.042		.039		.039	

+*p* < .10; *p < .05; ***p < .001

Table 3 presents the results for male and female prisoners who enter prison with less than a high school degree. As with the other analyses, we include prison-level fixed effects and sampling weights. Completing the requirements for a GED is associated with a lower score on the current morbidity scale for men (*b* = −.033, *p* < .05) but not women. Table 4 examines who participates in high school education classes while in prison in more detail. Less than half (43.5 %) of men who entered prison with less than a GED (*n* = 4,334) participated in a high school education class while in prison. There are 60 men in five prisons where no one with less than a GED reported participating in high school classes. It is possible that these prisons do not offer these types of courses so they are dropped from this descriptive analysis. Men who participate in high school education classes have a younger overall age and are overrepresented in the age 18 to 29 category. There are 1,837 men between the ages of 18 and 39 who entered prison with less than GED education and almost half of them (49.4 %) participated in high school education classes compared to only 41.1 % of the 2,083 men between the ages of 30 and 49 and 34.5 % of the 354 men aged 50 years or older.

**Table 3:** Results from Regressing Attaining a GED in Prison on Standardized Current Morbidity Scale for Men and Women Entering Prison with Less Than a GED with Prison-Level Fixed Effects

	Current Morbidity Scale			
	Men		Women	
	*B*	*p*	*b*	*p*
Attain a GED in Prison	−.033	*	−.065	
Age	.010	***	.016	***
Race/Ethnicity (White Ref.)				
Black	−.062	***	−.078	*
Latino	−.071	***	−.045	
Other	.011		−.053	
Employed Prior to Incarceration	−.023	+	−.027	
Marital Status (Never Married Ref.)				
Married	.036	*	.081	+
Widowed/Divorced/Separated	.019		.015	
Time Served to Date	.001		.002	
First time Incarcerated	−.001		−.058	*
Model Statistics				
Number of Inmates	4128		1102	
Number of Prisons	225		62	
Overall R Squared	.13		.11	
Rho	.078		.087	

+*p* < .10; *p < .05; ***p < .001

**Table 4:** Descriptive Analysis Examining Participation in High School Education Classes for Inmates who Enter Prison with Less Than a GED-Level of Education Stratified by Gender

	Men (*n* = 4,274)				Women (*n* = 2,781)			
	No Participation	Participate	*t/x* ^*2*^	*p*	No Participation	Participate	*t/x* ^*2*^	*p*
Age (se)	34.8 (.23)	32.4 (.23)	7.5	< .000	35.0 (.34)	33.6 (.42)	2.6	< .009
Age 18 to 29	930 (38.9)	907 (48.1)	36.0	< .000	201 (29.4)	181 (38.8)	11.2	< .001
Age 30 to 49	1226 (51.3)	857 (45.4)	14.7	< .000	449 (65.6)	265 (56.9)	9.1	< .003
Age 50 or older	232 (9.7)	122 (6.5)	14.6	< .000	34 (5.0)	20 (4.3)	.3	< .593
Race/Ethnicity								
White	606 (25.4)	548 (29.1)	7.2	<.007	220 (32.2)	161 (34.6)	.7	< .399
Black	1244 (52.1)	956 (50.7)	.8	<.362	304 (44.4)	189 (40.6)	1.7	< .191
Latino	421 (17.6)	274 (14.5)	7.5	<.006	116 (17.0)	80 (17.2)	.01	< .927
Other	117 (4.9)	108 (5.7)	1.5	<.229	44 (6.4)	36 (7.7)	.7	< .398
Marital Status								
Never Married	1510 (63.3)	1299 (68.9)	14.7	< .000	366 (53.6)	239 (51.2)	.5	< .489
Married	343 (14.4)	220 (11.7)	6.8	< .009	103 (15.1)	79 (17.0)	.8	< .376
Widowed/Divorced/Separated	535 (22.3)	366 (19.4)	5.3	< .021	214 (31.3)	146 (31.5)	.00	< .962
Employed Prior to Incarceration	1614 (67.7)	1239 (65.8)	1.8	< .176	336 (49.1)	240 (51.5)	.6	< .428
Years of Education Prior to Incarceration (se)	8.9 (.04)	9.2 (.04)	−4.6	< .000	9.1 (.07)	9.1 (.07)	.1	< .907
Time Served to Date (se)	4.0 (.11)	5.3 (.13)	−7.6	< .000	1.9 (.13)	3.0 (.20)	−5.0	< .000
First time Incarcerated	1068 (45.4)	899 (48.2)	3.4	< .067	389 (57.5)	277 (60.0)	.7	< .401
Prison Experience								
Violent Offender	1128 (47.2)	1072 (56.8)	38.9	< .000	161 (23.5)	169 (36.3)	22.0	< .000
Drug Offender	501 (21.0)	329 (17.4)	8.4	< .004	196 (28.7)	122 (26.2)	.9	< .357
Job Training Program Participation	429 (18.2)	38.4 (723)	214.2	< .000	104 (15.4)	150 (32.2)	44.9	< .000
Visits from Family/Friends Past Month	545 (23.2)	566 (30.0)	25.6	< .000	143 (21.2)	151 (32.4)	18.0	< .000
Phone Calls from Family/Friends Past Week	1910 (81.1)	1628 (86.4)	21.1	< .000	510 (75.7)	377 (80.9)	4.4	< .037
Written Up for Any Violation	1253 (53.4)	601 (32.0)	195.6	< .000	405 (60.2)	180 (38.6)	51.2	< .000

Compared to men who do not participate in high school education classes, men who participate are overrepresented by whites (29.1 % vs. 25.4 %, *p* < .01) and those who have never been married (68.9 % vs. 63.3 %, *p* < .001). Participants have a higher average number of years of school completed prior to entering prison (9.2 vs. 8.9, *p* < .001) and have been in prison longer (5.3 vs. 4.0, *p* < .001). Results from a multivariate analysis (not shown) confirm these findings. Men who participate in high school education classes are also more likely to be violent offenders (56.8 % vs. 47.2 %, *p* < .001) and less likely to be drug offenders (17.4 % vs. 21.0 %, *p* < .01). They are more likely to also participate in a job training program (38.4 % vs. 18.2 %, *p* < .001). These men seem to have higher levels of social support in the form of phone calls (86.4 % vs. 81.1 %, *p* < .001) and visits (30.0 % vs. 23.2 %, *p* < .001). Finally, they are less likely to be written up for a violation (32.0 % vs. 53.4 %, *p* < .001).

Forty-one percent of all women (*n* = 2,781) entered prison with less than a GED level of education. Among those women, 40.5 % participated in a high school education program with 16.9 % of participants obtaining a GED by the time of the interview. Similar to men, women who participate in high school education classes while in prison tend to be younger (33.6 vs. 35.0, *p* < .01). Of the 382 women who entered prison with less than a GED education between the ages of 18 and 29, 47.4 % participated in a high school education class. Thirty-seven percent of the 714 women aged 30 to 49 participated and 37.0 % of the women aged 50 or older participated. None of the demographic variables other than age are associated with participating in a high school education class; however, time spent incarcerated is positively associated (3.0 vs. 1.9, *p* < .001) which was also found in the multivariate analysis (not shown). The findings concerning the additional measures of the prison experience are similar to men. Violent offenders are more likely to participate in high school education classes (36.3 % vs. 23.5 %, *p* < .001); although being a drug offender is not significant. Female inmates who participate in high school education classes are also more likely to participate in job training programs (32.2 % vs. 15.4 %, *p* < .001) and to receive visits (32.4 % vs. 21.2 %, *p* < .001) and phone calls (75.7 % vs. 80.9 %, *p* < .05) from family and friends. Finally, participants are less likely to be written up for a violation (38.6 % vs. 60.2 %, *p* < .001).

Finally, the analyses thus far have assumed that each health condition is equal in its association with education, but this is likely not the case given different disease progressions and etiologies. Therefore, a supplementary analysis examines the relationship between education and hypertension more closely. Of all of the medical conditions included in this study, hypertension is most likely to be proximately influenced by education. Indeed it appears that education both prior to and during incarceration is negatively associated with hypertension for men. Controlling for age, race, marital status, years incarcerated and first incarceration episode with prison-level fixed effects, each year of education is associated with three percent lower odds (*p* < .05) of having lifetime hypertension for men. For those men entering prison with at least a GED, their odds of lifetime hypertension are decreased by eight percent (*p* < .10) controlling for all other factors.

We extend our analyses of current hypertension and prison education among only those men who enter prison without a GED or higher degree in order to reduce bias associated with time order. Using a fixed effects logistic regression model, we find that men entering prison with less than a GED level of education who participate in a high school education program while in prison have 19 % lower odds of reporting current problems with hypertension (*p* < .05)^2^ compared to those who did not participate in high school education classes conditioned on availability. For those who earned their GED while in prison, their odds of reporting current problems with hypertension are even lower (*OR* = .71; *p* < .05). If we reduce this sample to men who are less than 30 years of age (the age group most likely to earn a GED in prison), attaining a GED is associated with 56 % lower odds of reporting problems with current hypertension (*p* < .05).^3^ For women, years of education (*OR* .96, *p* = .247) and having a GED or higher (*OR* = .79, *p* = .163) prior to being incarcerated is not associated with lifetime hypertension. Neither participating in high school education classes (*OR* = 1.07, *p* = .840) nor obtaining a GED while in prison (*OR* = .87, *p* = .813) is associated with hypertension for women.

## Discussion

Our study demonstrates the importance of education for health among incarcerated adults. Previous research has documented the many benefits of education for inmates which extends to local communities [19]. While this study focuses on high school education, post-secondary education for inmates is also a growing concern. In 2015 the Obama administration and the U.S. Department of Education announced the Second Change Pell Pilot Program to test new models to allow inmates to receive Pell Grants and pursue the postsecondary education in order to “to create a fairer, more effective criminal justice system, reduce recidivism, and combat the impact of mass incarceration on communities” citing that “for every dollar invested in correctional education programs, four to five dollars are saved on three year re-incarceration costs” [20]. Our study suggests that improved health may be an additional benefit through potential increases in learned effectiveness, health literacy and ability to engage in health promotion, all of which has the potential to improve community health [21]. Although not the focus of our study, the findings also point to benefits in the prison experiences for inmates who participate in prison education classes including greater external social support and lower likelihood of receiving a violation. Given these multiple far-reaching benefits of education, prisons should consider expanding basic education for inmates. In this national sample, only about 40 % of inmates who entered prison with less than a GED-level of education participated in high school education classes by the time of the survey.

Our study is the first to demonstrate the generalizability of the education-health association beyond the non-institutionalized population. These findings are important because they support the idea that education is a “fundamental cause” of health [1] even among one of the most select groups in the United States in terms of both health and education. Our results are also consistent with previous research documenting the poorer health status of incarcerated women compared to men [11] and the gendered nature of the education-health association [22] where education serves as a protective resource more for women than for men. Further, although not the focus of this paper, it is important to highlight the relationship of education and health compared to the other demographic controls in the study. Specifically, while education operates in a comparable manner to other research of noninstitutionalized adults, the relationship between race and health and marital status and health both operate in directions that are opposite to general findings [23, 24]. Both black male and female prisoners have a lower cumulative morbidity count compared to white male and female prisoners and prisoners who are currently married have worse health compared to those who are not married. This is important because, again, it speaks to the highly select nature of the incarcerated population but it also indicates further evidence of the robustness of the education-health association.

There are two additional points to consider. First, a comprehensive meta-analysis conducted by the RAND Corporation [19] found that GED programs are the most common education programs in prison, yet all types of education programs available (i.e., GED, adult basic education, postsecondary, and vocational) notably reduce post-release recidivism. The report concluded, however, that data do not exist to evaluate dose–response effects or the specific program characteristics that benefit inmates. The findings from the current study suggest that when moving forward with stronger research designs, additional proximate and distal indicators of program efficacy, such as health-related outcomes, should be considered. These studies could also address causal ordering. In our study we conceptualized health as the outcome. But a more comprehensive study examining the life course of inmates can parse out the dynamic processes of education and health throughout the life span. It should also be noted that the data used in our study are from 2004 when educational programming was more widely available. The 2008 recession affected correctional programming leading to dramatic changes in the number of programs offered, the sizes of the classes, the modes of delivery, and the number of inmate participants [19]. It is possible that our findings are influenced by period effects, although, research has consistently documented an education-health association in the general population across time and cohorts.

Second, this paper discussed selection into prison as a function of gender and the gendered nature of the education-health association. We encourage future research to consider how race-based selection processes [25] influence education and health in prisons. Regardless of race, high school drop outs are five times more likely to go to prison than high school graduates [26] and national statistics show that blacks have among the lowest graduation rates for high school students [27]. The double disadvantage of race and class inequality is striking for incarceration rates. Over 16 % of black men without a high school degree entered prison annually from 1995 to 2001 compared to just 3.4 % of white men without a high school degree, and this disparity grew from previous time periods [26]. Other research has documented that the cumulative risk of imprisonment by age 34 for black men without a high school education is 68 % [28]. This translates into 27 % of white prison inmates having not completed high school or their GED compared to 44 % of black prison inmates [29]. Collectively, this research highlights the differential selection among racial and ethnic minorities into the prison system which may influence the education-health association.

### Limitations

This study has several limitations that need to be considered. First, the data are limited to inmates in state correctional facilities. While state prison inmates comprised 1.3 million of the 1.5 million prison inmates at midyear 2011 [30], it is important to consider that our findings may not be generalizable to all incarcerated persons, especially those in local county jails. Second, this study relies on self-reported health conditions. However, self-report data are an essential and commonly used source of health indicators in research [31], and the SISCF is the best data set available to answer the research question because it is the only large, nationally representative survey of inmates available in the United States. Third, the data are cross-sectional and do not provide information on onset of health conditions. That is, this study is unable to account for the timing of diagnosis or the severity of symptoms. This study is also limited by our inability to examine the neighborhood contexts that individuals are exposed to prior to prison. Such information may be particularly important since incarcerated persons are drawn from distinct geographic areas [32] referred to as prison “feeder communities” [33]. In other words, distinct sociodemographic communities bear the burden of mass incarceration including young poor people of color from disadvantaged neighborhoods [32, 34, 35]. Therefore, it is not clear whether the study findings reflect education as mitigating the deleterious effects of imprisonment, or a prior association between poor health, low levels of education, and high propensity for incarceration.

## Conclusion

Results from our study provide additional support for the notion that the association between education and health may be, in part, causally oriented. We do so by focusing on a highly select population and the gendered nature of the education-health association. We encourage future researchers to examine the proximate pathways through which this observed association may operate [36]. This is especially important considering that incarcerated persons comprise a vulnerable, disadvantaged, and largely unhealthy subset of the U.S. population who may be reflective of the larger marginalized segments of society. Our findings are timely as prisons are having to address correctional healthcare practices. The identified health promotion needs of prisoners include education in health and empowerment, support in adopting health behavior, development of life skills, and education related to specific illnesses, among others [37]. Our results support the notion that the provision of primary and secondary education to prisoners may be an important element for health promotion and increasing the life chances and longevity of prisoners after they are released back to their communities.

## Footnotes

1 The models were estimated using different functional forms of age including age^2^ and age^3^. For women, the coefficient remain unchanged (−.016). For men, the coefficient reduced slightly from−.038 to−.029. Given this, the most parsimonious model is presented.

2 The sample size for this model is 3,498; 531 observations were dropped from the fixed effects model because the prison did not have variation in the outcome variable (current hypertension).

3 The sample size for this model is 390; 786 observations were dropped from the fixed effects model because the prison did not have variation in the outcome variable (current hypertension).
